# Advanced age a risk factor for illness temporally associated with yellow fever vaccination.

**DOI:** 10.3201/eid0706.010605

**Published:** 2001

**Authors:** M Martin, L H Weld, T F Tsai, G T Mootrey, R T Chen, M Niu, M S Cetron, GeoSentinel Yellow Fever Working Group

**Affiliations:** Division of Bacterial and Mycotic Diseases, Centers for Disease Control and Prevention, Atlanta, Georgia 30333, USA.

## Abstract

In 1998, the Centers for Disease Control and Prevention was notified of severe illnesses and one death, temporally associated with yellow fever (YF) vaccination, in two elderly U.S. residents. Because the cases were unusual and adverse events following YF vaccination had not been studied, we estimated age-related reporting rates for systemic illness following YF vaccination. We found that the rate of reported adverse events among elderly vaccinees was higher than among vaccinees 25 to 44 years of age. We also found two additional deaths among elderly YF vaccinees. These data signal a potential problem but are not sufficient to reliably estimate incidence rates or to understand potential underlying mechanisms; therefore, enhanced surveillance is needed. YF remains an important cause of severe illness and death, and travel to disease-endemic regions is increasing. For elderly travelers, the risk for severe illness and death due to YF infection should be balanced against the risk for systemic illness due to YF vaccine.


### Advanced Age a Risk Factor for Illness Temporally Associated with Yellow Fever Vaccination

**Michael Martin,*† Leisa H. Weld,* Theodore F. Tsai,* Gina T. Mootrey,* Robert T. Chen,* Manette Niu,‡ Martin S. Cetron,* and the GeoSentinel Yellow Fever Working Group[1]**

*Centers for Disease Control and Prevention, Atlanta, Georgia, USA; †Emory School of Medicine, Atlanta, Georgia, USA; and ‡Center for Biologic Evaluation, U.S. Food and Drug Administration, Rockville, Maryland, USA

In 1998, the Centers for Disease Control and Prevention was notified of severe illnesses and one death, temporally associated with yellow fever (YF) vaccination, in two elderly U.S. residents. Because the cases were unusual and adverse events following YF vaccination had not been studied, we estimated age-related reporting rates for systemic illness following YF vaccination. We found that the rate of reported adverse events among elderly vaccinees was higher than among vaccinees 25 to 44 years of age. We also found two additional deaths among elderly YF vaccinees. These data signal a potential problem but are not sufficient to reliably estimate incidence rates or to understand potential underlying mechanisms; therefore, enhanced surveillance is needed. YF remains an important cause of severe illness and death, and travel to disease-endemic regions is increasing. For elderly travelers, the risk for severe illness and death due to YF infection should be balanced against the risk for systemic illness due to YF vaccine.

In 1998, the Centers for Disease Control and Prevention (CDC) was notified of severe illnesses occurring days after immunization with yellow fever (YF) vaccine in two elderly U.S. residents. Both vaccinees had been in good health, and neither was immunocompromised. One patient died. Because these cases were unusual and the risk for illness following vaccination with YF vaccine in the elderly had not been studied, we estimated age-related reporting rates for YF vaccine associated systemic illness.

YF is an acute febrile illness caused by a mosquito-borne flavivirus (1). Clinical presentation ranges from a mild, febrile illness to a life-threatening infection involving hepatic failure, renal dysfunction, myocardial injury, and a bleeding diathesis. YF is endemic in much of tropical South America and sub-Saharan Africa (2).

Two live, attenuated YF vaccines were developed in the early 1930s, the French neurotropic vaccine (FNV) and the 17D vaccine (1,3-5). Production of FNV was halted in 1982 because its neurotropism had resulted in cases of encephalitis, primarily among children (1,6,7). Derivatives of the 17D strain are the only YF virus strains currently used for vaccine production. These vaccines are not cloned from a single virus but consist of a heterologous population of virions (8). Human trials with the 17D YF vaccine in the 1930s found low rates of adverse events and protective levels of YF viral neutralizing antibodies in more than 95% of vaccinees (3,5). More recent studies have shown that protective antibodies may last 30 to 35 years (9).

Early field trials and experiments with the 17D virus demonstrated that virulence varied with the passage level. Some substrains were overattenuated and led to low rates of seroconversion, while others were associated with post-vaccine encephalitis (10,11). A seed lot system, which standardizes vaccine preparation and limits passage of the virus, was recognized as the production standard in 1945 (1,12). The World Health Organization publishes recommended standards. Previous reports of YF adverse events have focused primarily on hypersensitivity or neurologic sequelae. A review of reports submitted to the Vaccine Adverse Event Reporting System (VAERS) in the United States from 1990 to 1997 found a rate of probable anaphylaxis after YF vaccine immunization of 1 per 131,000 vaccine doses distributed

Address for correspondence: Martin Cetron, Division of Global Migration and Quarantine, National Center for Infectious Diseases, Centers for Disease Control and Prevention, Mailstop E03, 1600 Clifton Road, Atlanta, GA 30333, USA; fax: 404-498-1633; e-mail: Mcetron@cdc.gov

[1]Jeff Altman, University of Washington, Seattle, Washington; Vernon Ansdell, Kaiser Permanente, Honolulu, Hawaii; Elizabeth Barnett, Boston University, Boston, Massachusetts; Michele Barry, Yale University, New Haven, Connecticut; Bradley Connor, Cornell University, New York, New York; David Freedman, University of Alabama at Birmingham, Birmingham, Alabama; Alejandra Gurtman, Mount Sinai Medical Center, New York, New York; Elaine Jong, University of Washington, Seattle, Washington; Phyllis Kozarsky, Emory University, Atlanta, Georgia; Russell McMullen, University of Washington, Seattle, Washington; Jan Patterson, University of Texas, San Antonio, Texas; Bradley Sack, Johns Hopkins University, Baltimore, Maryland; Mary E. Wilson, Harvard University, Cambridge, Massachusetts; Martin Wolfe, Traveler's Medical Service of Washington, Washington, D.C.

(13). Since the seed lot system was introduced, 21 cases of post-vaccine encephalitis have been reported worldwide (20 patients recovered, one died) (1,14); 16 (76%) of these cases occurred in children <9 months. Meningoencephalitis has on rare occasions been reported among adults after immunization with the vaccine (15,16), and severe, multisystemic illness has recently been reported in seven YF vaccinees (17-19).

YF vaccine has had a long history of efficacy and presumed safety (1). Nonetheless, a reexamination of its safety profile has been prompted by its increased use in international travelers and by these recent reports of serious adverse events (17-19).

### Methods

Adverse events following vaccination with YF vaccine reported to VAERS were collected and categorized as systemic, nonsystemic, or unrelated and were classified by age group. The number of doses administered by age group (denominators) was estimated from the age distribution of travelers receiving the vaccine at a sample of travel clinics and from the number of doses distributed to civilians in the United States by the vaccine manufacturer. Reporting rates for systemic and nonsystemic adverse events were calculated by dividing the events reported by an estimate of the number of people receiving the vaccine in each age group.

### Source of Cases and Case Classification

VAERS, a passive surveillance system for adverse events, monitors vaccine safety in the United States and is jointly operated by CDC and the U.S. Food and Drug Administration (20). All civilian U.S. VAERS reports from 1990 through 1998 listing YF vaccine were reviewed. Reports of death, hospitalization or disability, a life-threatening illness, or illness requiring an emergency room or doctor visit were analyzed. Reports that did not involve any of these events were considered less serious and were excluded from analysis. Reports were blinded for age and reviewed independently by three physicians. Adverse events were classified as neurologic, multisystemic, uncomplicated neurologic or systemic, nonspecific, hypersensitivity, local reactions, or unrelated (Table 1). If more than one category was appropriate, the most serious category, in terms of reaction to the specific vaccine components, was selected. These categories were defined for the purposes of this study and reflect an interest in examining adverse events that might be related to the vaccine virus rather than those that might be immune responses to other vaccine components. The investigators reached a consensus on the categorization of each report before unblinding the ages.

A systemic adverse event (SyAE) was defined as a multisystemic (excluding anaphylactic) or neurologic reaction. Adverse events categorized as uncomplicated neurologic or systemic, hypersensitivity, or local reaction were defined as other adverse events (OAE). A second analysis used a more stringent definition of SyAE that included only neurologic or multisystemic cases requiring hospitalization or resulting in death (SyAE*).

VAERS reports that did not include the age of the vaccinee, provided another explanation of the adverse event (e.g., local reaction from another vaccination), or indicated inappropriate administration or inadvertent use (e.g., during pregnancy) were excluded. Reports from children <15 years of age and military personnel were excluded because no adequate estimates of the number of persons who received YF vaccine in these groups were available. As a comparison, similar analyses were done on adverse events after hepatitis A (HA) vaccine reported to VAERS during 1994 to 1998 (Table 1).

Table 1. Categories of vaccine adverse events[a]

**Neurologic (SyAE)**

- Guillain-Barré syndrome, new onset seizures, encephalitis, myelitis, altered mental status, focal cranial or peripheral neurologic deficits, paresthesias, vertigo, headaches (headaches alone are not sufficient for neurologic diagnosis)[b]
- Onset <2 weeks after vaccination
- Duration >72 hours

**Multisystemic (SyAE)**

- Myalgias, arthralgias, rhabdomyolysis, elevated transaminases, respiratory distress, nausea, vomiting, diarrhea, nephropathy, disseminated intravascular coagulation, +/-fever[b]
- Onset <2 weeks after vaccination
- Duration >72 hours

**Neurologic/systemic, uncomplicated (OAE)**

- Cases that met the neurologic or systemic criteria but had a full and rapid clinical recovery in <72 hours

**Nonspecific events without other focal finding (OAE)**

- Dizziness, headache
- Nausea, vomiting, or diarrhea alone

**Hypersensitivity (OAE)**

- Rash, urticaria, +/- fever
- Anaphylaxis, angioedema
- Onset within 48 hours of vaccination

**Local reaction (OAE)**

- Localized pain, swelling, erythema, or warmth (at injection site)
- Onset within 1 week of vaccination

**Unrelated to vaccine (excluded from AE analysis)**

- A clear, alternative diagnosis confirmed by laboratory criteria accounts for symptoms and signs; sometimes this is an underlying illness
- Another cause implied or stated in the physician's report
- For hepatitis A vaccine, onset of adverse event is >6 weeks

SyAE = systemic adverse event; OAE = other adverse events; AE = adverse event(s).
[a]Listed in order from most to least severe.
[b]Examples, but not limited to these signs, symptoms and conditions.

VAERS solicits reports not only of events known to be causally related to vaccine but also of all events temporally related to vaccination, some of which may be coincidental. Evaluating the causal relationship of an event to a specific vaccine may be also confounded by the routine practice of administering multiple vaccines at a single visit. Furthermore, VAERS has several other methodologic limitations inherent to passive surveillance systems, such as under-, biased-, and incomplete reporting and lack of consistent diagnostic criteria. Thus, VAERS reporting rates are, at best, a crude estimate of event rates. Given these limitations of passive surveillance systems, neither reporting rates nor the number of events reported to VAERS may automatically be considered synonymous with the incidence of adverse events. Elevated VAERS reporting rates may best serve as sentinel signals suggesting hypotheses to test in other more rigorous databases before definitive conclusions can be reached.

### Denominator

The sole manufacturer of YF vaccine in the United States (now known as Aventis Pasteur) provided the annual number of YF vaccine doses purchased by civilian providers from 1995 through 1998. The annual number of doses from 1990 through 1994 was extrapolated from the number of doses in 1995. We assumed that the number of doses increased each year at the same annual rate as occurred from 1995 to 1996. Doses for 1997 and 1998 were not used in the extrapolation because unusual supply and regulatory issues influenced the number of doses provided in these years. Telephone interviews with health-care providers indicated little or no waste of YF vaccine, which for civilian use in the United States is sold predominantly in single-dose vials. Thus, it was assumed that the total number of doses sold was a good estimate of the total number of doses administered.

The manufacturers of HA vaccine (Havrix, SmithKline Beecham, Rixensart, Belgium; and Vaqta, Merck & Co., Inc., West Point, PA) provided the annual number of doses of HA vaccine purchased from 1995 through 1998. We estimated that 10% of HA vaccine was wasted and that 50% of vaccinees received both doses in the series. The total number of doses sold was reduced by these amounts to estimate the total number of doses administered. These doses did not include those used for outbreak control.

Thirteen U.S.-based GeoSentinel clinics, which provide YF vaccine to international travelers, reviewed records of YF and HA vaccine administration. GeoSentinel is an international network of travel and tropical medical clinics established in 1995 as a collaborative effort by CDC and the International Society of Travel Medicine (21). GeoSentinel monitors geographic and temporal infectious disease trends among people crossing international borders; the clinics are chosen to detect sentinel events in travelers seen in clinics before and after travel. Within the United States, these clinics are considered representative of clinics that offer YF vaccine. All YF and HA vaccine recipients during the most recent 12-month period for which complete information was available were categorized by age group (15 to 24 years, 25 to 44 years, 45 to 64 years, 65 to 74 years, and ≥75 years). Data for children <15 years of age and military personnel were excluded because these groups are underrepresented at U.S. GeoSentinel clinics. Other than these exceptions, the age distribution for YF vaccine recipients at GeoSentinel clinics was assumed to represent national YF vaccine use. The age distribution for HA vaccine recipients from these clinics was also assumed to represent national use excluding the same exceptions and outbreak control.

The number of YF vaccine doses administered to each age group was estimated by multiplying the total number of YF vaccine doses per year by the proportionate age group distribution estimated from the GeoSentinel clinics. Age group-specific reporting rates for SyAE per 100,000 doses and reporting rate ratios for SyAE were calculated with a reference group of 25- to 44-year-old vaccine recipients. The 25- to 44-year-old group was chosen because of the previously reported increased risk for adverse events among younger YF vaccine recipients (1). Although the risk is highest for infants <4 months of age, it is unclear at what age the risk reaches a nadir. Confidence intervals were calculated based on standard statistical assumptions for confidence intervals (CI) for ratios of rates, although because of the limitations of passive surveillance systems, these assumptions may not hold.

### Results

From 1990 through 1998, VAERS received 166 reports of YF vaccine adverse events that met the criteria for review (Figure 1). Thirty-five (21%) of these reports were categorized as SyAEs and 36 (22%) as OAEs. Of the 10 VAERS reports for patients >65 years of age, one was categorized as an OAE and the other nine as SyAE. The latter included the two index patients, one additional death, and six patients with various signs and symptoms, including fever, headache, malaise, myalgia, nausea, somnolence, and ataxia. Two of these six patients were hospitalized. Ninety-five (57%)

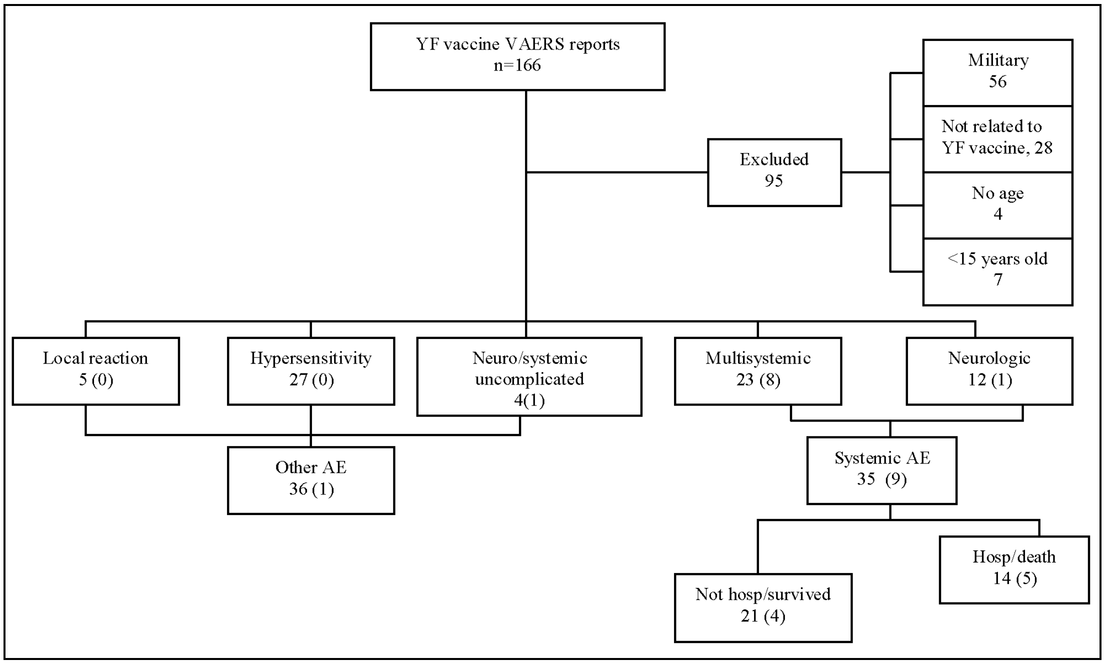

Figure 1. Diagram of yellow fever (YF) vaccine adverse events reports (1990-1998). n = number of reports; O = number of reports in patients >65 years old. VAERS = Vaccine Adverse Event Reporting System.

reports were excluded because they did not include the age of the vaccinee (4 reports), or the vaccine was used in military personnel (56 reports) or a child <15 years old (7 reports), or an alternative cause of the adverse event was reported (28 reports). Seventeen patients were hospitalized, and three patients, ages 63, 67, and 79 years, died. The clinical course for the two index patients and the two additional deaths was characterized by a nonspecific febrile syndrome with fatigue, myalgia, and gastrointestinal symptoms, rapidly progressing to a severe multisystemic illness with dysfunction of liver, kidneys, lungs, central nervous system, as well as thrombocytopenia, and possible disseminated intravascular coagulopathy (18).

From 1990 through 1998, U.S. civilian providers purchased an estimated 1.55 million doses of YF vaccine. The age distribution of YF vaccine recipients was estimated from 5,125 YF vaccine recipients in 13 GeoSentinel clinics. The reference group, ages 25 to 44 years, accounted for 45% of the sample; 285 (5.6%) of the vaccinees were 65 to 74 years of age, and 73 (1.4%) were ≥75 years of age (Figure 2).

The overall reporting rate for a SyAE after YF vaccination was 2.4 per 100,000 doses, and the reporting rate for death was 0.2 per 100,000 doses; for those ≥65 years of age, the overall reporting rate for SyAE was 8.3 per 100,000 doses, and the reporting rate for death was 1.8 per 100,000 doses. During this period, an estimated 108,000 doses were administered to those ≥65 years of age. The reporting rate for a SyAE for the reference age group (25 to 44 years) was 1.6 per 100,000 doses and increased progressively for each

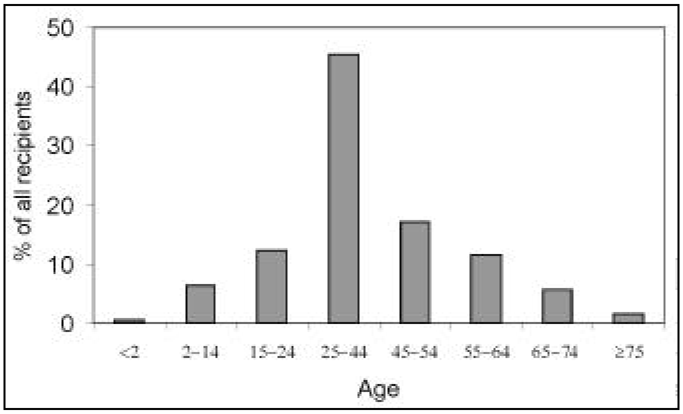

Figure 2. Estimated age range for YF vaccine recipients, n=5,125. Percentage of children ≤15 years of age is underestimated as these groups were excluded from analysis (see text).

older age group to 5.8 per 100,000 doses for vaccinees 65 to 74 years of age and 18.1 per 100,000 doses for those ≥75 years of age (Table 2). Compared with the reference group, the reporting rate ratio for an SyAE for those 65 to 74 years of age was 3.7 (95% CI 1.3-10.7); for those ≥75 years of age, it was 11.6 (95% CI 3.7-36.3). Conversely, the reporting rate for OAE decreased for each older age group.

When analysis of SyAEs was restricted to vaccinees who died or were hospitalized, the pattern was even stronger (Table 3). Compared with the reference group, the reporting rate ratio for an SyAE* for those 65 to 74 years of age was 12.3 (95% CI 2.0-73.2); for those ≥75 years of age, it was 31.8 (95% CI 4.5-225.9).

Of the 35 patients with SyAEs, 19 (54%) received at least one other vaccine in addition to YF vaccine. When analysis was restricted to those who received only YF vaccine, the reporting rate ratio for a SyAE* for those 65 to 74 years of age was 6.1 (95% CI 1.4-27.3); for those ≥75 years of age it was 23.9 (95% CI 5.3-106.6). Six of the nine patients ≥65 years of age with SyAEs received only YF vaccine, and all five patients ≥65 years of age who were hospitalized or died had received that vaccine alone.

During 1995 to 1998, VAERS received 310 reports of adverse events after immunization with HA vaccine that met the inclusion criteria. From an estimated 3.2 million doses of HA vaccine, 30 patients were hospitalized, and none died. This is double the total number of doses of YF vaccine and almost three times the number of YF vaccine doses given to people ages ≥75 years. The reporting rate for an SyAE after HA vaccine for vaccine recipients 65 to 74 years of age was 6.2 per 100,000. This was higher than the 2.5 per 100,000 reporting rate for the reference group (ages 25 to 44 years); however, there was no consistent increase in the reporting rate for SyAE for each older age group, and recipients ages ≥75 years did not have a different reporting rate from those ages 25 to 44 years (reporting rate ratio =1.9, 95% CI 0.6-6.3) (Table 4) (Figure 3).

### Discussion

Severe illness in two elderly recipients of YF vaccine, one of whom died shortly after immunization, prompted this collaborative study, which examined reporting rates for adverse events among elderly YF vaccine recipients in the United States (18). We found a higher reporting rate for SyAEs among elderly YF vaccine recipients than among YF vaccine recipients ages 25 to 44 years. This increase in reporting rates persisted when adverse events were limited

Table 2. Reporting rates (RR) and reporting rate ratios (RRR) for yellow fever (YF) vaccine adverse events by age, 1990–1998

| Age (years) | No. vaccine doses | No. OAE[a] | OAE reports/ 100,000 doses | RRR (95% CI) | No. SyAE[b] | SyAE reports/ 100,000 doses | RRR (95% CI) |
|---|---|---|---|---|---|---|---|
| 15-24 | 189,991 | 11 | 5.79 | 2.5 (1.2-5.5) | 3 | 1.58 | 1.0 (0.3-3.6) |
| 25-44 | 702,783 | 16 | 2.28 | Reference | 11 | 1.57 | Reference |
| 45-64 | 442,605 | 8 | 1.81 | 0.8 (0.3-1.9) | 12 | 2.71 | 1.7 (0.8-3.9) |
| 65-74 | 86,222 | 1 | 1.16 | 0.5 (0.1-3.8) | 5 | 5.80 | 3.7 (1.3-10.7) |
| ≥75 | 22,085 | 0 | 0 | undefined | 4 | 18.11 | 11.6 (3.7-36) |
| Total | 1,443,686 | 36 | 2.49 | | 35 | 2.42 | |

CI = confidence interval.
[a]OAE: other adverse event (uncomplicated neurologic/systemic, hypersensitivity, or local reaction).
[b]SyAE: systemic adverse event (multisystemic [excluding anaphylactic] or neurologic reaction)

Table 3. Reporting rates (RR) and reporting rate ratios (RRR) for serious systemic yellow fever (YF) vaccine adverse events (SyAE*) by age, 1990–1998

| Age (years) | No. vaccine doses | No. of OAE*[a] | OAE* reports/ 100,000 doses | RRR (95% CI) | No. SyAE*[b] | SyAE* reports/ 100,000 doses | RRR (95% CI) |
|---|---|---|---|---|---|---|---|
| 15-24 | 189,991 | 12 | 6.32 | 1.8 (0.9-3.5) | 2 | 1.05 | 3.7 (0.5-26) |
| 25-44 | 702,783 | 25 | 3.56 | Reference | 2 | 0.29 | Reference |
| 45-64 | 442,605 | 15 | 3.39 | 1.0 (0.5-1.8) | 5 | 1.13 | 4.0 (0.8-20) |
| 65-74 | 86,222 | 3 | 3.48 | 1.0 (0.3-3.2) | 3 | 3.48 | 12.3 (2.0-73) |
| ≥75 | 22,085 | 2 | 9.06 | 2.5 (0.6-10.7) | 2 | 9.06 | 32 (4.5-226) |
| Total | 1,443,686 | 57 | 3.95 | | 14 | 0.97 | |

CI = confidence intervals.
[a]OAE*= other adverse events (i.e., uncomplicated neurologic or systemic event; hypersensitivity; local reaction) OR systemic adverse events not requiring hospitalization or resulting in death.
[b]SyAE* = serious systemic adverse events, including only neurologic or multisystemic adverse events requiring hospitalization or resulting in death; this is distinguished from the term SyAE, which indicates all systemic adverse events (See Table 2).

Table 4. Reporting rates (RR) and reporting rate ratios (RRR) for hepatitis A vaccine adverse events by age, 1995–1998

| Age (years) | No. HA doses | No. OAE[a] | OAE reports/ 100,000 doses | RRR (95% CI) | No. SyAE[b] | SyAE reports/ 100,000 doses | RRR (95% CI) |
|---|---|---|---|---|---|---|---|
| 15-24 | 387,031 | 21 | 5.43 | 1.8(1.1-3.0) | 7 | 1.81 | 0.7 (0.3-1.6) |
| 25-44 | 1,444,895 | 44 | 3.05 | Reference | 36 | 2.49 | Reference |
| 45-64 | 1,096,391 | 23 | 2.10 | 0.7 (0.4-1.1) | 26 | 2.37 | 1.0 (0.6-1.6) |
| 65-74 | 241,453 | 4 | 1.66 | 0.5 (0.2-1.5) | 15 | 6.21 | 2.5(1.4-4.6) |
| ≥75 | 61,760 | 1 | 1.62 | 0.5 (0.1-3.9) | 3 | 4.86 | 1.9 (0.6-6.3) |
| Total | 3,231,530 | 93 | 2.88 | | 87 | 2.69 | |

HA = hepatitis A [vaccine]; CI = confidence intervals.
[a]OAE = other adverse events (uncomplicated neurologic/systemic, hypersensitivity, or local reaction).
[b]SyAE: systemic adverse event (multisystemic [excluding anaphylactic] or neurologic reaction)

to patients who required hospitalization or died and when recipients who also received other vaccines were excluded. Although we did find an elevated rate of reported SyAEs following HA vaccine in the 65- to 74-year-old group, we did not find a similar increase in persons ≥75 years, despite almost three times as many doses sold overall and twice as many adverse events reported to VAERS for all age groups combined. No deaths following HA vaccination were reported.

Our analysis showed that the reporting rate for systemic illness requiring hospitalization or leading to death after YF vaccination was 3.5 per 100,000 among people 65 to 75 years of age and 9.1 per 100,000 for people ≥75 years. For a rough comparison, the risk for vaccine-associated paralytic poliomyelitis due to oral polio vaccine was estimated as 1 per 2.5 million (22,23). A review of a passive surveillance system in the United Kingdom that receives reports from primary-care physicians also found a similar increase in SyAEs among elderly YF vaccine recipients (unpub. data).

Close examination of the two index cases and two additional deaths that followed YF vaccination shows four cases with similar clinical presentations, all of which share important characteristics with viscerotropic wild-type YF infection (18). Clinical presentations were characterized by fever, myalgia, headache, and confusion rapidly progressing to a multisystemic illness and death in three of the patients. The vaccine strain of YF virus was isolated from the serum of two patients and the cerebrospinal fluid of one. Sequence analysis of these isolates and the elevated antibody titers suggest an overwhelming infection caused by the selective amplification of a mutated virus subpopulation. The temporal relationship between severe illness and YF vaccination, the similar clinical presentations, and the laboratory results favor the hypothesis that these adverse events are causally related to YF vaccine. Recently, three cases of similar systemic adverse events after YF vaccination resulting in death were reported: two from Brazil, patients ages 5 and 22 years (Brazilian 17DD vaccine) and one from Australia, patient 56 years old (17D-204 vaccine) (17,19).

An increased risk for severe disease due to the vaccine strain of *Yellow fever virus* among older YF vaccine recipients is biologically plausible. Numerous reports and studies have shown that deaths and severe illnesses occur more frequently among the elderly with other flaviviral infections (e.g., West Nile encephalitis, Japanese encephalitis, Saint Louis encephalitis, Murray Valley encephalitis, and tick-borne encephalitis), while these infections are more likely to be self-limited in children (24-27). Similarly, investigations of YF outbreaks in the 1930s and 1940s found increased case-fatality rates among the oldest patients (28-30).

This study has several limitations. Rates calculated from VAERS data have to be interpreted with caution because of the problems inherent in a passive reporting system. Estimates of adverse events based on VAERS reports are likely to underestimate actual events (31). Our data,

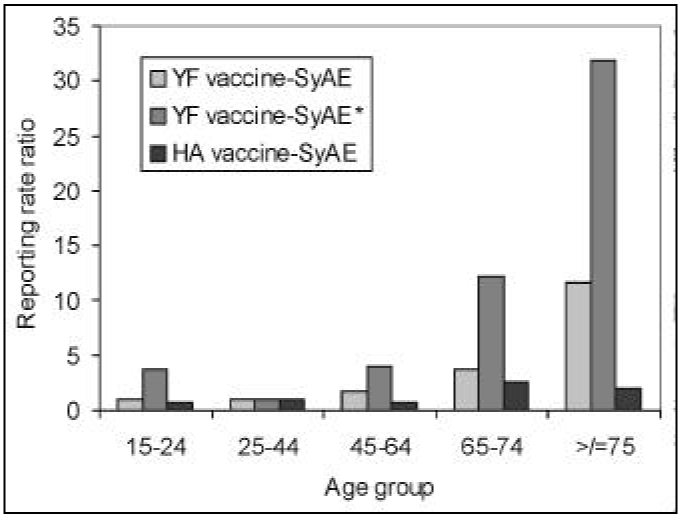

Figure 3. Reporting rate ratios for systemic adverse events (SyAE) and serious adverse events (SyAE*) after yellow fever (YF) vaccination and hepatitis A (HA) vaccination.

therefore, may reflect minimum estimates of these adverse events. Age-related reporting bias may also have influenced our results. Age-related reporting bias would decrease the significance of the reporting rate ratios only if SyAEs, particularly those leading to hospitalization and death, among vaccinees <65 years of age were reported less often than among vaccinees ≥65 years of age.

Another important limitation is that the estimated age distribution of travelers from the GeoSentinel clinics receiving YF vaccine in 1998 was assumed to apply for the entire study (1990 to 1998) and to be generalizable to the entire United States. However, if as many suspect, the proportion of elderly travelers has increased in recent years, this extrapolation will have overestimated the number of older travelers and have the effect of underestimating the reporting rate and reporting rate ratio of adverse events in the elderly. We excluded data on children <15 years of age, which caused a slight, proportionate increase in the denominator for the remaining vaccine recipients and a slight underestimate of the reporting rate for adverse events in all other age groups. Also, the calculation of denominators relied on assuming that the increase in vaccines administered between 1995 and 1996 held for 1990 to 1994.

An additional limitation is that age-specific SyAEs to YF vaccine may reflect an age-related response to vaccines in general or an increased amount of background illness in the elderly. Analysis of VAERS reports for SyAEs to HA vaccine did not support this conclusion; however, HA vaccine is an inactivated vaccine, and another live, attenuated vaccine would have served as a better control. We attempted to look at adverse events reported for oral typhoid vaccine, but the limited number of VAERS reports precluded a quantitative analysis.

Finally, although severe illness occurred days after YF vaccination in the cases we investigated, this temporal association does not prove causality. Definitive clinical or pathologic evidence identifying YF vaccine as the cause of severe illness or death for most cases reported to VAERS is lacking and is not routinely part of this surveillance system.

YF remains an important cause of severe illness and death in tropical South America and sub-Saharan Africa. In recent years, *Aedes aegypti*, the mosquito vector of urban YF, has reestablished itself in South America, increasing the likelihood of large, explosive outbreaks, and in both South America and sub-Saharan Africa, the number and size of outbreaks have increased in the last 20 years (32-34). Concomitant with these changes in the distribution of the vector and ongoing outbreaks, travel from the United States to disease-endemic regions has increased substantially (35). Quantitative risk assessments of YF among travelers to disease-endemic areas have not been done; however, the risk for acquiring YF has been highlighted by the recent deaths of four unvaccinated travelers due to YF imported to Europe and the United States (36-40).

The 17D YF vaccine has a long history of reported safety and efficacy and has played an important role in YF control, one of the public health triumphs of the 20th century. Age-specific recommendations and production standards for this important vaccine have been modified as a result of safety and efficacy issues that have become apparent with increased use (1,2,4,41,42). These modifications have preserved the vaccine as a vital tool for disease prevention and control. Defining the risk for adverse events among elderly vaccine recipients is an extension of these important efforts.

### Conclusion

This study provides data quantifying the relative reporting ratios of SyAE following YF vaccination among people ≥65 years of age in the United States. However, several issues must be addressed before any changes or restrictions to YF vaccine recommendations are proposed.

These SyAEs are still relatively rare (2.4 per 100,000 doses) in the United States, where an estimated 200,000 doses are given annually. The risk for unvaccinated travelers acquiring YF remains undefined; so risk-benefit estimates of YF vaccine are difficult to develop. In the absence of this important information, we suggest the following steps. Our observations should be confirmed by studies in different populations. Enhanced surveillance for systemic adverse events following YF vaccination should be introduced at U.S. certified vaccination centers and in other countries where YF vaccine is used. This enhanced surveillance should be combined with prospective follow-up that includes appropriate clinical, epidemiologic, and laboratory assessments of cases, biologic specimens, and vaccine or vaccine lots. In addition, epidemiologic studies should be designed to explore both host- and vaccine-specific factors associated with systemic adverse events (43).

In the interim, elderly YF vaccine recipients and their health-care providers should be cautioned about the possible risks of vaccination. Travel itineraries should be scrutinized, and the vaccine given only to those traveling to areas that report YF or are in the YF-endemic zone.

YF causes serious, life-threatening infections, and the vaccine is highly effective. The virus is responsible for substantial morbidity and death in disease-endemic areas and until more definitive evidence of vaccine-related adverse events is accumulated, the benefit-risk ratio of mass vaccination in YF-endemic countries favors continuation of a universal vaccine policy under the Expanded Programme on Immunization. Meanwhile, efforts to enhance our understanding of both the risks and benefits of YF vaccine and refine its use to maximize its safety and effectiveness should be accelerated.

### Acknowledgments

We thank Stefanie Steele and Laura Letteau for their assistance in data collection; Alice Brebner, Betsy Abraham, Angie Bricco, and Mike Bloh for help in getting denominator data; Scott Kellerman for his assistance with the hepatitis A analysis; and John McGowan for reviewing the manuscript.

Dr. Martin is an Epidemic Intelligence Service Officer at the Centers for Disease Control and Prevention. He works in the Division of Bacterial and Mycotic Diseases.

### References

1. Monath TP. Yellow fever. In: Plotkin SA, Mortimer EA, Orenstein W, editors. Vaccines. 3rd ed. Philadelphia: WB Saunders; 1999. p. 815-79.
2. Centers for Disease Control and Prevention. Health information for international travel 2000-2002. Atlanta: U.S. Department of Health and Human Services; 1999.
3. Theiler M, Smith HH. The use of yellow fever virus modified by in vitro cultivation for human immunization. J Exp Med 1937;65:787-800.
4. Barrett ADT. Yellow fever vaccines. Bull Inst Pasteur 1987;85:103-24.
5. Smith HH, Penna HA, Paoliello A. Yellow fever vaccination with cultured virus (17D) without immune serum. Am J Trop Med 1938;18:437-68.
6. Stones PB, MacNamara FN. Encephalitis following neurotropic yellow fever vaccine administered by scarification in Nigeria: epidemiological and laboratory studies. Trans R Soc Trop Med Hyg 1955;49:176-86.
7. Eklund CM. Encefalitis infantil en Costa Rica y Honduras despues del empleo de la vacuna Dakar contra la fiebre amarilla. Boletin Oficina Sanitaria Panamericana 1953;35:505-16.
8. Ryman KD, Xie H, Ledger TN, Campbell GA, Barrett ADT. Antigenic variants of yellow fever virus with an altered neurovirulence phenotype in mice. Virology 1997;230:376-80.
9. Poland JD, Calisher CH, Monath TP, Downs WG, Murphy K. Persistence of neutralizing antibody 30–35 years after immunization with 17D yellow fever vaccine. Bull World Health Organ 1981;59:895-900.
10. Fox JP, Penna HA. Behavior of 17D yellow fever virus in rhesus monkeys; relation to substrain, dose, and neural or extraneural inoculation. Am J Hyg 1943;38:152-72.
11. Theiler M. The virus. In: Strode G, editor. Yellow fever. New York: McGraw-Hill; 1951. p. 39-136.
12. United Nations Relief and Rehabilitation Administration (UNRRA). Standards for the manufacture and control of yellow fever vaccine. Epidemiological Information Bulletin 1945;1:365.
13. Kelso JM, Mootrey GT, Tsai TF. Anaphylaxis from yellow fever vaccine. J Allergy Clin Immunol 1999;103:698-701.
14. Fatal viral encephalitis following 17D yellow fever vaccine inoculation. JAMA 1966;198:671-2.
15. Merlo C, Steffen R, Landis T, Tsai TF, Karabatsos N. Possible association of encephalitis and 17D yellow fever vaccination in a 29-year-old traveler [letter]. Vaccine 1993;11:691.
16. Drouet A, Chagnon A, Valance J, Carli P, Muzellec Y, Paris JF. Méningo-encéphalite après vaccination anti-amarile par la souche 17D: deux observations. Rev Med Interne 1993; 14:257-9.
17. Vasconcelos PFC, Luna EJ, Galler R, Silva LJ, Coimbra TL, Barros VLRS, et al. Serious adverse events associated with yellow fever 17DD vaccine in Brazil: a report of two cases. Lancet 2001;358:91-7.
18. Martin M, Tsai TF, Cropp B, Chang GJ, Holmes DA, Tseng J, et al. Fever and multisystemic organ failure associated with 17D-204 yellow fever vaccination: a report of four cases. Lancet 2001;358:98-104.
19. Chan RC, Penney DJ, Little D, Carter IW, Roberts JA, Rawlinson WD. Hepatitis and death following vaccination with 17D-204 yellow fever vaccine. Lancet 2001;358:121-2.
20. Chen RT, Rastogi SC, Mullen JR, Hayes SW, Cochi SL, Donlon JA, et al. The Vaccine Adverse Event Reporting System (VAERS). Vaccine 1994;12:542-50.
21. Freedman DO, Kozarsky PE, Weld LH, Cetron MS. GeoSentinel: the global emerging infections sentinel network of the international society of travel medicine. J Travel Med 1999;6:94-8.
22. Strebel PM, Sutter RW, Cochi SL, Biellik RJ, Brink EW, Kew OM, et al. Epidemiology of poliomyelitis in the United States one decade after the last reported case of indigenous wild virus-associated disease. Clin Infect Dis 1992;14:568-79.
23. Prevots DR, Sutter RW, Strebel PM, Weibel RE, Cochi SL. Completeness of reporting for paralytic poliomyelitis, United States, 1980 through 1991. Arch Pediatr Adolesc Med 1994;148:479-85.
24. Tsai TF. Flaviviruses. In: Mandell GL, Bennett JE, Dolin R, editors. Principles and practices of infectious diseases. 5th ed. Philadelphia: Churchill Livingstone; 2000. p. 1714-36.
25. Centers for Disease Control and Prevention. Outbreak of West Nile-like viral encephalitis—New York, 1999. MMWR Morb Mortal Wkly Rep 1999;48:845-9.
26. Hayes CG. West Nile fever. In: Monath TP, editor. The arboviruses: epidemiology and ecology. Vol 5. Boca Raton (FL): CRC Press; 1989. p. 59-82.
27. Tsai TF, Popovici F, Cernescu C, Camplbell GL, Nedelcu NI. West Nile encephalitis epidemic in southeastern Romania. Lancet 1998;352:767-71.
28. Hanson H. Observations on the age and sex incidence of deaths and recoveries in the yellow fever epidemic in the department of Lambayeque, Peru, in 1921. Am J Trop Med Hyg 1929;9:233-9.
29. Beeuwkes H. Clinical manifestations of yellow fever in the West African native as observed during four extensive epidemics of the disease in the Gold Coast and Nigeria. Trans R Soc Trop Med Hyg 1936;30:61-86.
30. Kirk R. An epidemic of yellow fever in the Nuba mountains, Anglo-Egyptian Sudan. Ann Trop Med Parasitol 1941;35:67-108.
31. Rosenthal S, Chen R. The reporting sensitivities of two passive surveillance systems for vaccine adverse events. Am J Public Health 1995;85:1706-9.
32. Robertson SE, Hull BP, Tomori O, Bele O, LeDuc JW, Esteves K. Yellow fever: a decade of reemergence. JAMA 1996; 276:1157-62.
33. Van der Stuyft P, Gianella A, Pirard M, Cespedes J, Lora J, Peredo C, et al. Urbanisation of yellow fever in Santa Cruz, Bolivia. Lancet 1999;353:1558-62.
34. World Health Organization. Yellow fever in 1987. Wkly Epidemiol Rec 1989;64:37-43.
35. World Tourism Organization. Yearbook of tourism statistics. 50th ed. Madrid: World Tourism Organization; 1998.
36. World Health Organization. Yellow fever in a traveler. Wkly Epidemiol Rec 1996;71:342-3.
37. McFarland JM, Baddour LM, Nelson JE, Elkins SK, Craven RB, Cropp BC, et al. Imported yellow fever in a United States citizen. Clin Infect Dis 1997;25:1143-7.
38. Barros MLB, Boecken G. Jungle yellow fever in the central Amazon [letter]. Lancet 1996;348:969-70.
39. Teichmann D, Grobusch MP, Wesselmann H, Temmesfeld-Wollbruck, Breuer T, Dietel M, et al. A haemorrhagic fever from the Côte d'Ivoire. Lancet 1999;354:1608.
40. Centers for Disease Control and Prevention. Fatal yellow fever in a traveler returning from Venezuela, 1999. MMWR Morb Mortal Wkly Rep 2000;49:303-5.
41. Fox JP, Lennette EH, Manso C, Aguiar JRS. Encephalitis in man following vaccination with 17D yellow fever virus. American Journal of Hygiene 1942;36:117-42.
42. Centers for Disease Control and Prevention. Yellow fever vaccine: recommendations of the Immunization Practices Advisory Committee (ACIP). MMWR Morb Mortal Wkly Rep 1990;39(RR-6):1-6.
43. Centers for Disease Control and Prevention. Fever, jaundice, and multiple organ systsem failure associated with 17D-Derived yellow fever vaccination, 1996-2001. MMWR Morb Mortal Wkly Rep 2001;50:643-5.
